# Single Molecule Methods for Monitoring Changes in Bilayer Elastic Properties

**DOI:** 10.3791/1032

**Published:** 2008-11-03

**Authors:** Helgi Ingolfson, Ruchi Kapoor, Shemille A. Collingwood, Olaf Sparre Andersen

**Affiliations:** Department of Physiology and Biophysics, Weill Cornell Medical College of Cornell University

## Abstract

Membrane protein function is regulated by the cell membrane lipid composition.  This regulation is due to a combination of specific lipid-protein interactions and more general lipid bilayer-protein interactions. These interactions are particularly important in pharmacological research, as many current pharmaceuticals on the market can alter the lipid bilayer material properties, which can lead to altered membrane protein function.   The formation of gramicidin channels are dependent on conformational changes in gramicidin subunits which are in turn dependent on the properties of the lipid.  Hence the gramicidin channel  current is a reporter of altered properties of the bilayer due to certain compounds.

**Figure:**
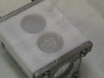


## Protocol

Please visit Springer Protocols for more information about preparing artifical bilayers and using gramicidin channels for probing membrane elasticity.

